# Predicting Epileptic Seizures in Advance

**DOI:** 10.1371/journal.pone.0099334

**Published:** 2014-06-09

**Authors:** Negin Moghim, David W. Corne

**Affiliations:** Heriot-Watt University, Edinburgh, United Kingdom; Kaohsiung Chang Gung Memorial Hospital, Taiwan

## Abstract

Epilepsy is the second most common neurological disorder, affecting 0.6–0.8% of the world's population. In this neurological disorder, abnormal activity of the brain causes seizures, the nature of which tend to be sudden. Antiepileptic Drugs (AEDs) are used as long-term therapeutic solutions that control the condition. Of those treated with AEDs, 35% become resistant to medication. The unpredictable nature of seizures poses risks for the individual with epilepsy. It is clearly desirable to find more effective ways of preventing seizures for such patients. The automatic detection of oncoming seizures, before their actual onset, can facilitate timely intervention and hence minimize these risks. In addition, advance prediction of seizures can enrich our understanding of the epileptic brain. In this study, drawing on the body of work behind automatic seizure detection and prediction from digitised Invasive Electroencephalography (EEG) data, a prediction algorithm, *ASPPR* (Advance Seizure Prediction via Pre-ictal Relabeling), is described. ASPPR facilitates the learning of predictive models targeted at recognizing patterns in EEG activity that are in a specific time window in advance of a seizure. It then exploits advanced machine learning coupled with the design and selection of appropriate features from EEG signals. Results, from evaluating ASPPR independently on 21 different patients, suggest that seizures for many patients can be predicted up to 20 minutes in advance of their onset. Compared to benchmark performance represented by a mean S1-Score (harmonic mean of Sensitivity and Specificity) of 90.6% for predicting seizure onset between 0 and 5 minutes in advance, ASPPR achieves mean S1-Scores of: 96.30% for prediction between 1 and 6 minutes in advance, 96.13% for prediction between 8 and 13 minutes in advance, 94.5% for prediction between 14 and 19 minutes in advance, and 94.2% for prediction between 20 and 25 minutes in advance.

## Introduction

Epilepsy is a neurological disorder, which affects 50 million people worldwide. It can be managed in some patients using prescription drugs. The remaining 20–30%, however, are likely to have a relapse after the initial remission, and some may develop drug resistant epilepsy [1]. Patients with uncontrolled epilepsy can be affected by accidents caused by unforeseen seizures as well as sudden unexpected death. They may also suffer from a multitude of other unwanted side effects such as memory loss, depression and other psychological disorders [2].

Despite the design of new anti-epileptic drugs, drug resistant epilepsy still lacks an ultimate solution [1]. Resective surgery, where the part of the brain that causes the seizures is removed [3], can only be applied to a small fraction of drug-resistant patients, the outcome of which is highly unpredictable. Additionally, the cause of drug-resistance is unknown. Resistance to the traditional AEDs in addition to the lack of effective seizure management treatments for this large population of patients demands newer, more effective seizure control therapies.

Electroencephalography (EEG) records electrical activity along the scalp, via the placement on the scalp of multiple electrodes; it measures voltage fluctuations resulting from ionic current flows within the brain. The time series of such voltage fluctuations (signals) recorded by the EEG is believed to correspond to neural activity, and, by comparing and contrasting EEG records from multiple patients, the EEG can therefore assist in discovering and characterizing abnormal activity in the brain [4]. The EEG is a very powerful diagnostic tool for many neurological disorders, epilepsy in particular. It can be used to distinguish between epileptic and non-epileptic seizures, via expert analysis of EEG to find patterns corresponding to ‘inter-ictal epileptiform discharges’ that are prevalent in epileptic patients but rare otherwise [3]. EEG can provide information about the location of the brain where the abnormality is created, and also can be used for identifying the type of epilepsy syndrome [4].

In most diagnostic and treatment-monitoring settings, the non-invasive EEG is used in the form of a scalp EEG. This form of EEG is however susceptible to low resolution of recordings and as a result may miss out on underlying epileptic patterns. Invasive EEG is used when non-invasive methods result in poor localisation [5]. One setting in which an invasive EEG serves to be more informative is pre-surgery evaluation for accurate localisation of the seizure-focus [5].

With the wide use of digital EEG recording tools, these kinds of data are becoming increasingly accessible for electronic manipulation. While EEGs were formerly used as a diagnosis and treatment specification tool for patients, access to the digitised form of this information has helped generate new fields of research, from neonatal seizure detection to understanding how the seizure unfolds in the epileptic brain.

In recent years, there has been growing research interest in seizure detection and prediction from EEG recordings. Being able to predict seizures, and couple this information with state of the art technology, will allow patients to take action prior to the occurrence of the seizure, minimizing potential risk [4].

Prior to the occurrence of seizures, a number of clinical symptoms have been shown to exist. These symptoms include increases in oxygen availability, cerebral blood flow, blood-oxygen-level-dependent signals, and changes in heart rate [6]–[9]. In addition to these changes, it is believed that an increased number of critical interactions among the neurons in the focal region unfold over time. This concept has allowed researchers to study EEGs in an alternative way, in order to find correlates of such processes and identify the pre-ictal (pre-seizure) state.

The main question researchers have been addressing is whether characteristic features can be extracted from an EEG which correlate with the occurrence (and time of the occurrence) of seizures. In that case, treatments could move from therapeutic and long-term preventive plans to on-demand strategies (i.e. immediately before the seizure occurs). In this context, Stein et al. [10] have envisioned the use of fast-acting anticonvulsant substances, while Fisher [11] has proposed deep-brain stimulation technology in order to reset the brain as soon as seizure activity is detected, to avoid the occurrence of seizures.

There is also the question of how a seizure occurs: is it a result of a sudden transition, or a gradual change, in the dynamics of the brain? The latter can be predicted through dynamics and is more likely the case for focal epilepsies, whereas the former is impossible to predict through dynamics and is more likely to be the case in general epilepsy [12].

The first attempts at seizure detection and prediction were carried out by Viglione and Walsh [13] in order to find seizure precursors using linear approaches for absence seizure EEGs (an ‘absence seizure’ is a category of seizure associated with brief loss of consciousness). Rogowski et al. [14] and later Salant et al. [15] were able to find changes 6 seconds before seizure onset, using an autoregressive model of the neuronal activity. Siegel et al. [16] found changes among 1-minute epochs prior to the seizure, and conducted further analysis on the spike occurrence rates in the EEG, indicating decreased focal spike-rate along with an increased rate of bilateral spikes before the seizure. This was followed by Le Van Quyen et al. [17] who compared pre-seizure dynamic variations to those of inter-ictal (between seizures) EEG and discovered a dynamical similarity index which seemed to decrease before seizures. Another groundbreaking discovery was made by Iaesemidis et al. [18] using the Lyapunov exponent and an open window analysis, revealing chaotic behaviour in invasive EEG and a decrease in this behaviour before the seizure.

Litt et al. [19] conducted a controlled experiment on continuous multi-day EEG recordings of a population of 5 patients evaluated for epilepsy surgery. This study reported that quantitative signal changes were detected 7 hours, 2 hours and 50 minutes prior to the seizure onset, with an increase in accumulated energy 50 minutes prior to the seizure onset, suggesting that the cascade of electrophysiological events, which have evolved from several hours before the onset, can be identified as a reliable and timely indication of seizures. The optimistic findings of this study were, however, not reproducible in later studies [20]. Moreover, the analytical approach used in this study did not lend itself to the development of a potential automated prediction-based treatment. Some other studies used an algorithmic approach on similar multi-day EEG recordings. Iasemidis et al. [21] reported, on unseen test data, Sensitivity 81% and Specificity 78%, for an average of 45.3 minutes in advance, on a dataset of only two patients. The method used in Iademidis et al's study is an algorithmic real-time statistical method which continuously calculates only a single feature (the short-term maximum Lyapunov exponent) and monitors T-index curves of this measure, and produces an alarm, if and when the measure exceeds a threshold. The same research group reported (in Chaovalitwongse et al. [22]) a Sensitivity of 68% and a Specificity of 85% for an average prediction window of 72 minutes in advance of seizures, when the same algorithm was tested on a population of 10 patients; the low Sensitivity of 68% had a large standard deviation of 24.42%, indicating that the method performed poorly on 20% of the patients The methods proposed by these studies, when tested on a larger dataset, led to poor results and were deemed unsuitable for real-life implementation.

Costa et al. [23] have tested various neural networks for classifying EEG records into one of four classes: ictal, pre-ictal, inter-ictal, and post-ictal. Ictal corresponds to the seizure activity, pre-ictal corresponds to the 300 seconds leading up to the occurrence of a seizure, post-ictal corresponds to the 300 seconds immediately following a seizure, and inter-ictal to the period between post-ictal and pre-ictal. They used 14 features for classifying the EEG signals, based on signal energy attributes, wavelet transforms and non-linear system dynamics. They carried out their study on EEG recordings of two patients from the Freiburg EEG Database [24].

Costa et al.'s study [23] reported Sensitivity 98.5%, Specificity 99.5% and Accuracy 98.5%; such high values in comparison with previous work arguably highlight the value of feature engineering and machine learning algorithms in individualised seizure classification. However, this experiment was only run on a small population of two patients, with limited independent repeats, and insufficient detail to allow full replication, so the generalisability of this level of performance in seizure classification is therefore still to be assessed.

It is helpful to distinguish *seizure detection* studies from *seizure prediction* studies. Seizure detection refers to the automatic recognition of seizures shortly before or after the actual onset, commonly in a short prediction window of a few seconds long. Seizure prediction represents the automatic recognition of seizures well in advance of the actual onset where the prediction window can be several minutes long [1]. The distinction between the two is important because their target application scenarios, and hence, the potential treatment strategies facilitated, are likely to be different. The results reported for detection are typically, and understandably, higher than those reported for prediction; this is simply because detecting an imminent seizure is easier than predicting it several minutes in advance.

A related distinction can be made in terms of the focus of a study. There are broadly two types: analysis-oriented, and prediction-oriented. In analysis-oriented studies, the focus is on analyzing the statistical properties of seizures. Typically, distinct characteristics of the EEG are evaluated in a retrospective manner, for their capability to discriminate between known ictal (seizure) and non-ictal (non-seizure) states of the brain. These studies are mainly aimed at exploratory analysis of the seizure state, involving the quantification of various statistical and other metrics of EEG that correlate with seizures [25], [26]. In prediction-oriented studies, the focus is on developing a predictive algorithm that might be a candidate for intervention-based treatment. In such studies, more attention is therefore paid to the use of features that can be quickly computed from streaming EEG data, and to the design of predictive algorithms [21], [22], [23]. The present study draws from analysis-oriented studies with regard to choosing features which are associated with good discrimination of the seizure state, and follows other prediction-oriented studies in terms of standards and protocols for method and evaluation. However, the present work goes beyond other prediction-oriented studies in terms of the coupling of a relatively long advance-prediction window (20 to 25 minutes) with validation of our approach over a relatively large sample (21 patients).

In this study, an advance prediction algorithm, ASPPR (Advance Seizure Prediction via Pre-Ictal Relabeling), is described. To use ASPPR for a specific patient, an initial data procurement and annotation stage is necessary, whereby at least 24 hrs of EEG data are recorded for that patient. A clinical expert then annotates the data, and ASPPR is employed to learn predictive models from that data; depending on the available computing hardware, learning the predictive models may take 1 or 2 hours. The resulting predictive models will be capable of operating in microseconds, and can be used for real-time intervention on that patient (provided it is installed on an appropriate device capturing data from sensors worn by that patient). ASPPR learns several distinct predictive models for a given patient, each associated with a ‘time-in-advance’ parameter *t*, indicating that the model attempts to predict that seizure onset will occur between *t* and *t*+5 minutes from now. In this study, results are reported for the 21 models (from *t* = 0 to *t* = 20 minutes, in steps of 1 minute). Independent applications of ASPPR are conducted on data from each of 21 patients in the Freiburg EEG database [24]. Following procurement of a patient's data and the subsequent annotation of ‘ictal’ regions in the data by a suitable expert, the ASPPR method comprises three main components: i) The feature selection component, which selects 14 out of 204 features for each patient, according to the ranking criteria of the ReliefF [27] feature selection algorithm. (The reason for selecting 14 in this study is given later in the ‘ReliefF’ subsection); ii) a simple data preparation step is performed to generate a separate dataset for training each specific ‘time-in-advance’ predictive model. iii) each individual ‘time-in-advance’ predictive model is trained, using a multi-class Support Vector Machine (multi-class SVM) [28]; in the present study, this training employs 10-fold cross validation on a randomly identified 70% of the an individual patient's data; the reported results indicate performance on the remaining 30% not used in training. Further, results for each individual patient are averaged over ten independent repeats, each involving a different randomized 70%/30% split.

Results are reported regarding the performance of this algorithm on each of the 21 patients represented in the Freiburg EEG dataset [24], and contrasted with benchmark performance measures obtained for each of the 21 patients by using Costa et al's [23] proposed 14 features to predict seizure onset (i.e. using ASPPR for the *t* = 0 ‘time-in-advance’ model only, and omitting the feature selection component). The ASPPR method consistently outperforms the benchmark (which has an S1-Score – harmonic mean of Sensitivity and Specificity - of 90.6% for predicting seizure onset between 0 and 300 seconds in advance), and achieves mean S1-Scores ranging from a minimum of 93.79% (for prediction between 10 and 15 minutes in advance) to a maximum of 96.30% (for prediction between 1 and 6 minutes in advance), and above 94% for several other ‘time-in-advance’ regimes, including: 96.13% for prediction between 8 and 13 minutes in advance; and 94.2% for prediction between 20 and 25 minutes in advance.

## Materials and Methods

### Source Dataset

The Freiburg EEG Database is one of the most cited resources used in modern seizure detection and prediction experiments. It is also one of the few publicly available invasive EEG datasets. The database contains 24 hour-long continuous pre-surgical invasive EEG recordings of 21 patients suffering from epilepsy, during which time several seizures are triggered and recorded. The patients vary widely in age, seizure type and seizure locality, but all suffer from focal medically intractable epilepsy and were admitted for pre-surgical evaluation at the Epilepsy Centre of the University Hospital of Freiburg, Germany. The retrospective evaluation of data received prior approval by the Ethics committee, Medical Faculty, University of Freiburg. Informed consent was obtained from each patient [24], [29].

Prior to public release, the Freiburg team prepared the data as follows. The data were recorded at a 256 Hz sampling rate, using the Neurofile NT digital EEG over 128 channels. From these electrodes, 6 channels were extracted by visual analysis of EEG experts, 3 of which were in the epilepsy focal area of the brain. The channels were labelled from 1–6, where channels 1–3 corresponded to focal recording and 4–6 comprised extra-focal recordings. The locations of these channels varied for each patient. Two types of signal files were prepared per patient: Ictal and Inter-ictal. Each Ictal files contain an hour of EEG signals per patient. There are typically three or four Ictal files per patient, each representing a one-hour window straddling a single seizure event. Hence, Ictal files hold ictal signals (corresponding to the seizure itself, typically around 180 seconds) as well as pre-ictal (signals from the 300 seconds immediately preceding a seizure), post-ictal signals (from the 300 seconds immediately following an ictal section) and inter-ictal signals (signals from all other time windows – i.e. not within a seizure or within 300 seconds either before or after a seizure).

**Figure 1:**
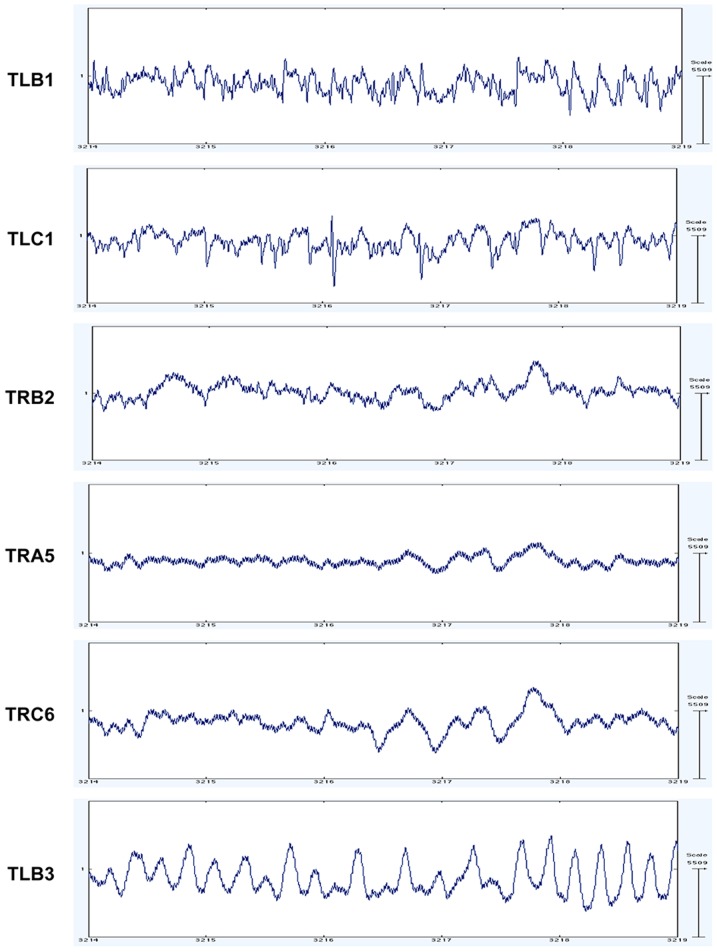
Invasive EEG recording of patient 2 from the Freiburg EEG Database. The image corresponds to pre-seizure and seizure data. Each row displays one of the 6 channel recordings. The name of the relevant EEG channel is listed to the right of each signal. The EEG signals were visualised using EEGLAB software [30].

The data files are in ASCII format and contain the EEG time series signals for 6 channels. The dataset comes with information on electrode specifications of the 6 channels and seizure onset and offset markers for all patients. Figure 1 displays EEG signals for Patient 2 from the Freiburg EEG Database.

### Data Preparation

This section clarifies how the Ictal files from the Freiburg database were treated to produce datasets for use in the ASPPR experiments. In short: (i) to reduce the time needed to build predictive models, only Ictal files were used (omitting between 19 and 22 hours of data available per patient that contained only EEG data further than 30 minutes from a seizure event); (ii) artefacts in the data were handled precisely as described in the notes accompanying the Freiburg database; (iii) a total of 204 feature time series were extracted for each patient (34 distinct features for each of the 6 EEG channels), using EEGLAB [30]; (iv) datasets were prepared comprising a time series of feature vectors for each patient, with each data instance (at a given time point) labeled appropriately as either *ictal*, *pre-ictal*, *inter-ictal* or *post-ictal*. The final prepared datasets for all patients from the Freiburg database can be accessed at https://code.google.com/p/asppr/. Fuller detail on the data preparation steps are provided next.

### Data Sampling

In the Freiburg EEG Database, both ‘Ictal’ and ‘Inter-ictal’ files are provided. Only data from the time-periods represented by the Ictal files were used. The Inter-ictal files only include non-seizure data and hence may not contain traces of seizure activity. By using only the data from the Ictal files, computational expense was significantly reduced without omitting pre-ictal and ictal data. The complexity of Support Vector Machine (SVM) learning is 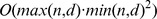 [31], where *n* is the number of instances in the training set and *d* is the number of features per instance. In the present context, *d* is always smaller than *n*, and Ictal files typically represented 3 hours of the total of 24 hours of data available per patient. Using the Inter-ictal files would therefore typically raise *n* by a factor of 8, increasing training time 8-fold.

### Artefact Removal

Artefacts in EEG recordings are forms of outliers and are considered as disturbances in a measured brain-signal, not originating from the brain. The different sources of artefacts are classified to external and internal categories. External artefacts result often from unsatisfactory technology such as exceeding measurement range of signals and disconnection of the electrode box. Internal artefacts arise from body activities that are either due to movements or bioelectrical potentials. The potential between electrodes changes as a result, from effects such as eye movement or muscular activity, causing an artefact [25].

When dealing with EEG data, the common practice of dealing with artefacts [25] is by visual detection and their subsequent removal with the aid of a capable software package. This practice was followed by the Freiburg team, whereby visual detection of artefacts was done by EEG experts. However rather than providing data with such artefacts already removed, the Freiburg database provides artefact specification data to accompany each patient's files. In this study, these pre-determined artefact specifications were followed, and artefacts subsequently removed using tools provided in the EEGLAB Matlab software package [30].

### Feature Engineering

Following artefact removal, features were then extracted from each Ictal file of each patient, in order to build datasets for training the predictive models. In total, 204 features were extracted, comprising 34 distinct features calculated independently for each of the 6 EEG channels. Extraction of a feature from an Ictal file corresponded to generating a time series of feature values, where the feature value at time *t* was calculated for each feature in the way described below, from the signal values within a time window ending at *t*. Following feature extraction, these 204 time series were converted into a dataset by exporting the vector of feature values for each time *t* starting from *t* = 5 seconds and continuing in steps of 5 seconds until the end of the ictal file was reached. Hence, for example, one hour of EEG data corresponded to 720 such data instances, each with 204 features. The first 14 features, used only for experiments to establish benchmark results, were drawn from the feature-engineering approach taken by EPILAB [33], [34] and also by Costa et al. [23]. Additional features were then extracted (an additional 20 features per channel) to support ASPPR's construction of more effective predictive models via feature selection from a richer feature set.

The 14 features used in Costa et al. [23] are divided into three general categories: signal energy, wavelet transform and non-linear dynamics.

### Features based on Signal Energy

Signal energy and accumulated energy are commonly employed features in EEG studies. A signal energy feature is typically the mean of signal energy over a given period [35], which can be expressed as in equation (1). 
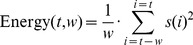
(1)where 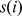
denotes the signal's amplitude at time-point *i*, and the signal values are sampled in sequence starting at time-point 1 and ending at time-point *w*.

**Figure 2:**
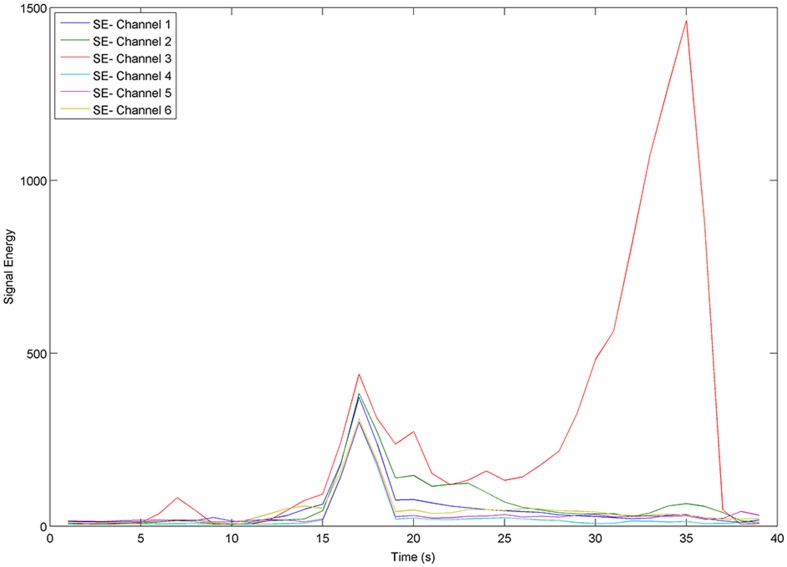
Signal Energy over 6 EEG channels for patient 2 from the Freiburg EEG Database. There is ictal activity from seconds 5 through 35. SE stands for Signal Energy.

Figure 2 displays the Signal Energy of patient 2 from the Freiburg EEG Database, captured from all 6 channels. From Figure 2, it can be observed that the signal energy produced by each channel is particularly variable during the seizure.

**Figure 3:**
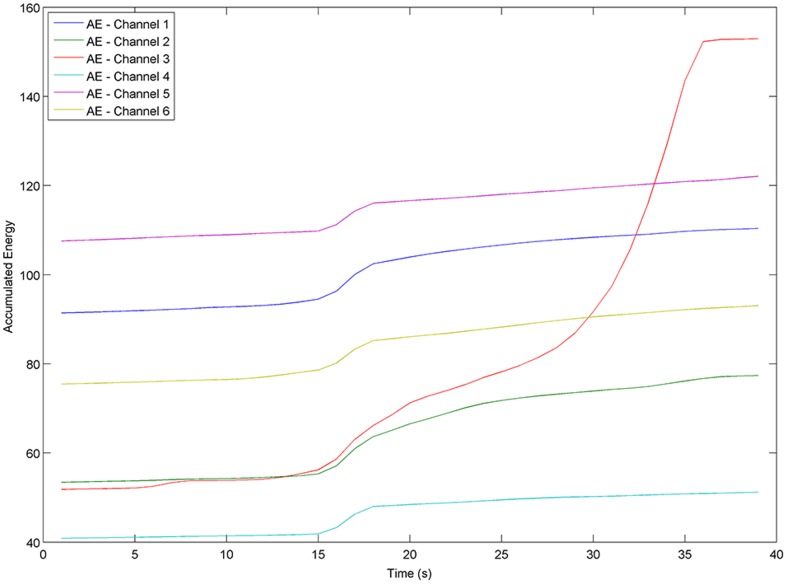
Accumulated Energy over 6 EEG channels for patient 2 from the Freiburg EEG Database. There is ictal activity from seconds 5 through 35. AE stands for Accumulated Energy.

Accumulated Energy (AE) is another powerful means of finding abnormal behaviour in the brain, and commonly employed in prediction-oriented seizure studies. AE in this article is the sum of successive values of signal energy from a series of moving windows, as expressed in equation (2), 
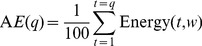
(2)where *AE*(*q*) indicates the accumulated energy at time-point *q*, calculated from successive values of signal energy via equation (1). Figure 3 displays the changes in accumulated energy of patient 2, calculated for each of the 6 EEG channels. The image displays similar trends of accumulated energy through different seizure-states for most channels. Channel 3 however displays a noticeably different trend for both signal energy (as seen in Figure 2) and accumulated energy. In potential intervention-based therapy, the *AE* value would be regularly reset by the recording device in use, most likely following a seizure episode, so that *AE* correlates with the energy accumulated since the most recent seizure. Since *AE* values are therefore not precisely known for the chronologically first seizure of each patient in the Freiburg database (24% of the seizures in the database), it is sensible to avoid bias by adding a suitable random constant (a different constant per file) to the *AE* values in each Ictal file (further details are provided with the engineered dataset available at the link indicated above).

In this study, ‘Signal energy’ (also called ‘signal level’ in Costa et al. [23]) was calculated for *w* = 1280, corresponding to 5 seconds at the 256 Hz sampling rate of the raw data. Following Costa et al. [23], two further signal energy features were also extracted, corresponding to signal energy (equation (1)) averaged over different windows. These were short-term energy (STE), where *w* = 2304 (9 seconds), and long-term energy (LTE) where *w* = 46080 (180 seconds).

### Features based on Discrete Wavelet Transforms

A Discrete Wavelet Transform (DWT) decomposes a signal, as does a Fourier Transform, but in a way that is able to reflect both frequency and temporal location properties of the signal, thereby potentially capturing characteristics that may have been missed by other features [36]. In particular, Fourier transforms are less able to capture temporal localization properties. The DWT is done with reference to a ‘mother wavelet’, which essentially controls the fine detail of the composition. In feature extraction for EEG, DWT is typically used to extract the signal energy component of a specific frequency band over a specific time-window. Following Costa et al. [23], we extracted eight features based on the DWT, corresponding to the signal energy for four frequency bands at each of two time windows. Once a component was extracted for a specific frequency band, equation (1) was applied to this signal component for a specific time window. The frequency bands extracted were 0 Hz–12.5 Hz, 12.5 Hz–25 Hz, 25 Hz–50 Hz and 50 Hz–100 Hz, and the time windows were ‘STE’, where *w* = 2304 (9 seconds), and ‘LTE’, where *w* = 46080 (180 seconds). The Daubechies mother wavelet [37] was used, with decomposition level 4 using the EEGLAB tool [30].

### Features based on non-linear dynamics

Non-linear features have had mixed review in the EEG signal-processing community. In some studies they have been suggested to be superior in performance in comparison to the linear features due to the aperiodic and unpredictable behaviour of seizures [17], [18], while other studies suggest that linear attributes perform as well, if not better than non-linear dynamics [25]. Non-linear features are drawn from the theory of dynamical systems [38]–[40] in contrast to the direct derivation of linear methods from the time-series signal. Non-linear dynamical systems can represent chaos, a perceivably unpredictable behaviour that is fundamentally deterministic. Dynamical systems capture the behaviour of a system in different states in time through fixed deterministic rules, and the states at any given time are derived from a state space. In this article, we used two features based on non-linear dynamics, namely the maximum Lyapunov exponent and the correlation dimension.

Lyapunov exponents [41], [42] formally relates to the rate of separation of infinitesimally, close trajectories in the phase space (i.e. the space where all possible states of the system are represented). Essentially they characterize the chaotic dynamics of a system, and the ‘maximal Lyapunov exponent’ serves as a surrogate measure for the stability of the system.

The correlation dimension [43] provides an alternative measure related to stability; it is an estimate of the number of active degrees of freedom of random points within a state space, and is calculated using the correlation integral [44]. Extraction of both the maximal Lyapunov exponent and the correlation dimension was done in this work by using the corresponding functionality in TSTOOL (a software package for analysing time-series data) [45], parameterized to sample each of these in windows of length 1280 (5 seconds).

### Additional Features: Moments, Spectral Band and Spectral Frequency

In addition to the 14 features above (used for recent prediction-oriented seizure studies and consequently underpinning our benchmark comparison), a further 20 distinct features were extracted per EEG channel. The first 6 of these further 20 features comprised the standard statistical measures of mean, skewness, and kurtosis of the raw signal value, averaged over STE and LTE windows (9 seconds and 180 seconds respectively). These represent the first, third and fourth standard statistical moments; note that the existing ‘signal energy’ feature already captures variance, which is the second moment.

A further ten features were extracted based on Spectral Band Power (SBP). SBP is simply the signal energy in a specific frequency range, as calculated via a Fourier transform. The ten SBP features used in this study correspond to the ten combinations arising from five frequency bands and the familiar two windows, STE (9 seconds) and LTE (180 seconds). The five frequency bands are chosen according to their common usage in analysis of neuronal signals since they seem to capture useful information [25], and are 0.5 Hz–4 Hz, 4 Hz–8 Hz, 8 Hz–13 Hz, 13 Hz–30 Hz, and 30 Hz–48 Hz; these bands are respectively termed *α*, *β*, *γ*, *δ*, and *ε*. SBP feature extraction was implemented using SBP functions In EEGLAB [30].

Finally, four features were extracted relating to Spectral Edge Frequency (SEF) [32]. SEF is a measure that characterises the signal's energy distribution in terms of how signal power is concentrated in the frequency spectrum. The measure SEF-*X* measure indicates the lowest frequency *F* such that *X*% of the spectral power is contained within the frequency band 0.5 Hz–*F* Hz. In this paper we use four features that comprise SEF-90 and SEF-50 (also called the median frequency), each measured over both the 9-seconds STE and 180-seconds LTE windows, and implemented using SEF functions available in EEGLAB [30].

### Labeling of Data Instances

Following the feature extraction phase, each data instance (recall: a data instance is a vector of features associated with a specific time-point) was labeled as one of the following states:

***Ictal*** **.** This labels the seizure activity in the brain and is marked precisely by EEG experts. It is of varying length but is typically close to 3 minutes long.

***pre-ictal*** **.** *pre-ictal* is marked as the 5 minutes immediately prior to the seizure onset and is believed to hold predictive markers of seizure activity [46], [47].

***post-ictal*** **.** *post-ictal* is marked as brain activity following the seizure offset for a duration of 5 minutes. Abnormal excitement in the signals may be observed in this state, particularly as patients are recovering from the seizures.

***inter-ictal*** **.** non-seizure data preceding the pre-ictal state and proceeding the post-ictal state are marked as inter-ictal, where studies have traced early predictors of future seizure activity [48]–[50].

**Figure 4:**
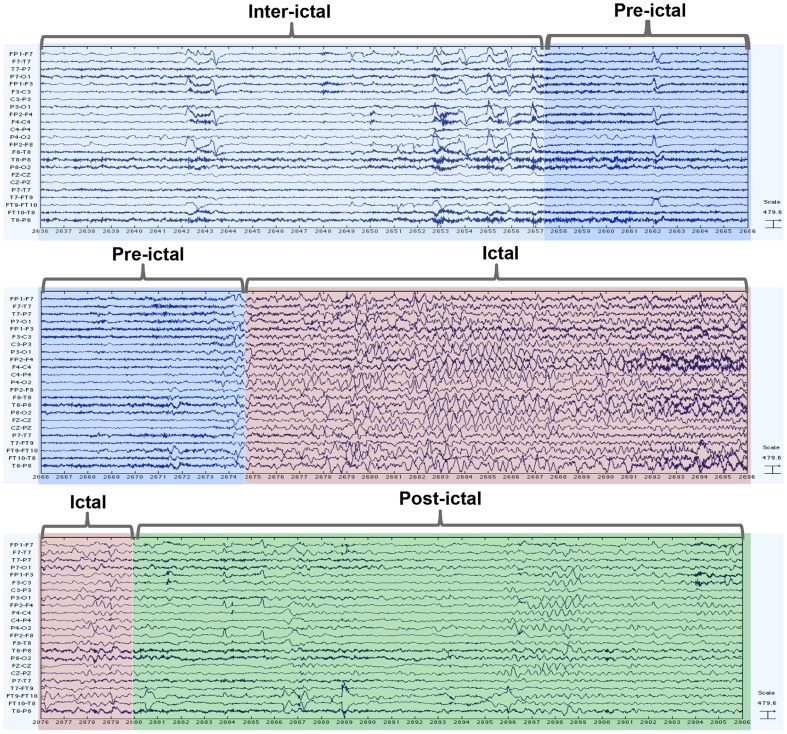
An annotated epoch of the Invasive EEG of an epileptic seizure. All four states of ictal, pre-ictal, ictal, post-ictal and inter-ictal are colour coded. EEG signals belong to patient 2 from the Freiburg EEG Database and were visualised using the EEGLAB software [30].

Figure 4 further illustrates the signal divisions of the EEG signals of a patient from the Freiburg EEG database. These labels were manually set according to the seizure onset and end markers provided in the Freiburg EEG Database notes. In training the predictive models, we use 4 states rather than 2 states as practiced in [23], since intuition suggests that distinguishing the activity among the four states will benefit the learning process, and facilitate a more even balance that avoids dominance of the inter-ictal states over ictal activity. When reporting test set results, however, these are always calculated on a 2-state basis (the ‘positive’ state being associated with a specific time-period in advance of a seizure).

Finally, the data labeling step described here was used to produce the dataset for training the ‘*t* = 0’ predictive models – in other words, models that attempt to distinguish pre-ictal instances from others, where a pre-ictal instance corresponds precisely to the expectation that seizure onset will occur between 0 and 5 minutes in the future. To produce training data for the ‘*t* = *N*’ predictive models, where *N* ranged from 1 minute to 20 minutes in steps of 1 minute, specific and simple manipulation was applied to the ‘*t* = 0’ dataset, which is detailed later.

### Advance Prediction Experiments: ASPPR

**Figure 5:**
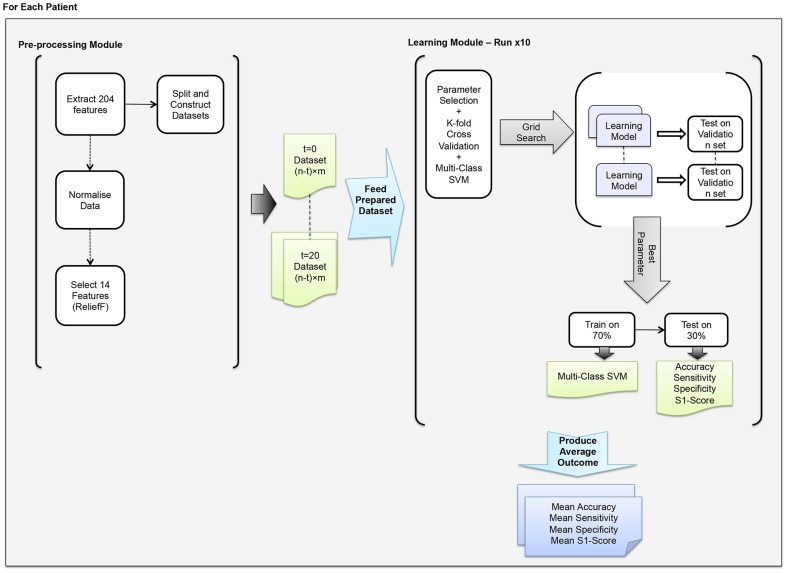
The architecture of ASPPR. The system consists of a Pre-processing Module and a Learning Module. The data preparation and initial experimental setup takes place in the Pre-processing Module, which varies for each experiment. This is separated from the learning and classification task in the Learning Module.

In total, 441 ‘advance prediction’ experiments are summarized in this article. Following a feature selection stage for each individual patient (see Figure 5), these experiments comprise the training and evaluation of 21 distinct ‘time-in-advance’ predictive models (from ‘*t* = 0’ to ‘*t* = 20’) for each of the 21 distinct patients represented in the Freiburg EEG database. Each of these experiments comprised ten independent trials, each of which was identical except for a different random split of the data into a training-set (70% of the instances) and a test set (30% of the instances). Experiments were parallelised over a cluster of thirty 8-core 64bit CentOS machines using Matlab parallel pooling [51].

### ReliefF

ReliefF [27] is a feature estimator algorithm, used for feature selection within ASPPR. ReliefF is a multi-class version of the original and well-known Relief algorithm [52]. ReliefF starts by assigning a weight of zero to each feature, and then works by iterating the following: a random data instance is sampled, and a sample of the closest data instances from the same class, and the closest data instances from other classes, are found. Calculations are then applied to adjust the feature weights, serving to enhance the relative weights of features that seem more important for discrimination between classes. A clear description of the ReliefF algorithm may be found in Robnik-Šikonja and Kononenko [53]. The output of ReliefF is a vector of feature weights, which is then used straightforwardly to rank the features in order of importance. ReliefF is stochastic, and therefore every time feature ranking is performed it is repeated ten times independently, and the overall feature ranking is based on the mean weights emerging from these ten trials. The computational complexity of ReliefF is suitable for large datasets, being *O*(*n*×*f*) [53], where *n* is the number of data instances and *f* is the number of features.

For each patient, prior to building the predictive models for that patient, ReliefF is used to find the 14 highest-ranked from the total of 204 extracted features; this set of 14 features is taken forward into the Learning module (Figure 5). The extraction of feature-sets of size 14 was done to facilitate comparison with benchmark results, which use a specific 14-feature subset from a previous study. In this way, better (or worse) performance can clearly be attributed to the choice of features themselves, rather than a function of the size of the feature set. However, without this constraint, optimized feature sets for different patients may well be of varying size; this is a topic for future work.

It was found that this set of 14 features varied both within and between patients; that is, for an individual patient, the top-14 features tended to vary across the ten independent trials of the feature selection stage. Similarly, feature-sets would typically be different for different patients. These observations are further discussed later in this article.

### Dataset Relabeling for Advance Prediction Experiments

The data instance labeling procedure described earlier led to a dataset for each patient that was used directly for training the time-in-advance ‘*t* = 0’ predictive models. To produce datasets for the other 20 predictive models for each patient, for ‘*t* = 1’ through to ‘*t* = 20’, this dataset was altered in the way described next. To produce the dataset for training the ‘*t* = *N*’ predictive model, the ‘*t* = 0’ dataset was amended in such a way that data labeled ‘*pre*-*ictal*’ corresponded to data instances from the time window beginning at *N*+5 minutes before seizure onset, and ending at *N* minutes before seizure onset. In this way, prediction of ‘pre-ictal’ from the subsequently trained model would correspond to predicting that a seizure would arise between *N* and *N*+5 minutes in advance. Amendment of the ‘*t* = 0’ dataset to obtain the ‘*t* = *N*’ dataset therefore consisted simply of (i) removing the data instances corresponding to the time window beginning at *N* minutes before seizure onset and ending at the point of seizure onset, and (ii) assigning the label ‘pre-ictal’ to all data instances corresponding to the time window beginning at *N*+5 minutes before seizure onset and ending at *N* minutes before seizure onset. Note that this corresponds to changing the labels (from *inter-ictal* to *pre-ictal*) of between 12 and to 60 data instances, and removing between 60 and 1200 data instances depending on the value of *N*.

**Figure 6:**
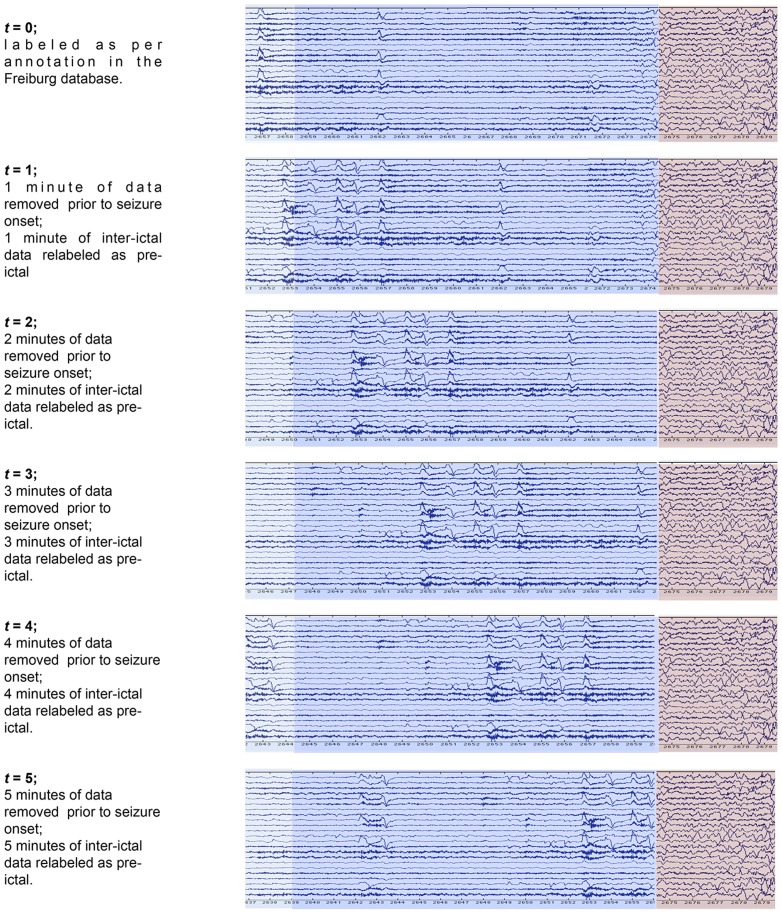
Amendment of datasets for time-in-advance predictive models. The top image displays a standard Ictal file where the ictal data is immediately preceded by a 300-seconds period of pre-ictal data, and also represents the dataset used to build ‘*t* = 0’ predictive models. The subsequent series of images then illustrates, from top to bottom, the datasets used to build the ‘*t* = 1’, ‘*t* = 2’, ‘*t* = 3’, ‘*t* = 4’ and ‘*t* = 5’ predictive models, constructed from the ‘*t* = 0’ model via removal of *t* minutes of immediately pre-ictal data, and relabeling (as pre-ictal) *t* minutes of inter-ictal data.

Figure 6 illustrates this relabeling process, showing how an ictal file is treated for six cases, ranging, top to bottom, from the ’*t* = 0’ case through to the ‘*t* = 5’ case. The ‘*t* = 0’ case shows the unaltered ictal file, with areas annotated as *pre-ictal* coloured blue, and *ictal* areas coloured lilac. In subsequent cases the figure illustrates the removal of *t* minutes of data immediately prior to the seizure onset, and the corresponding re-labeling of *t* minutes of inter-ictal data as pre-ictal.

### The Learning Module: Multi-Class Support Vector Machine

A support vector Machine (SVM) learns a classification model by transforming the instance-space (via a kernel function) and finding an optimal separating hyperplane in the transformed space [28], [54], [55]. The hyperplane derived by an SVM is such that it maximizes the distance (so-called ‘margin’) from the hyperplane to the closest transformed data instances at either side of the plane (the support vectors), while maximizing the extent to which data instances with different class labels are separated by the hyperplane. More formally, an SVM solves a two-class machine learning task involving *n* instances of training data 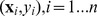, where each 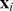 is a feature vector and 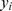, which is either −1 or 1, is its class label. Conceptually, the SVM first applies a transformation 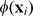 to every data instance 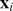, mapping the data into a space of higher dimensionality than the original feature space. The following optimization problem is then solved:
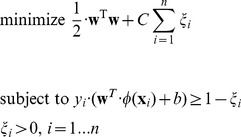
(3)where **w** is weight vector in the transformed space, *b* is a scalar (the equation 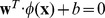 defines the desired hyperplane), *C* is a regularisation parameter, and the 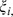
are non-negative ‘slack variables’. Without the slack variables (equivalently, with all the slack variables set at zero), the SVM is termed ‘hard-margin’, and the constraint in equation (3) forces all instances with label 1 to be mapped to one side of the hyperplane, and all instances with label −1 to be mapped to the other side. With slack variables allowed to be nonzero, the SVM is denoted ‘soft-margin’, and the formulation can cope with misclassifications (that is, the constraint can be satisfied for some collection of 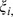 values). Minimization of the upper expression in equation (3) promotes generalization performance by keeping weight values low, and also minimizes the degree to which misclassified instances are on the wrong side of the hyperplane. The transformation 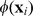
is ‘conceptual’ in the sense that it is never directly applied. Instead, a kernel function *K* is used, such that:
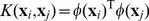
(4)


By using the kernel function directly, and exploiting the fact that the optimization process only requires the calculation of dot-products between transformed instances, SVM training can be done with reasonable computational cost despite relying on potentially very-high dimensional transformations.

ASPPR uses a Multi-class SVM classifier, implemented here by using the MC-SVM function from the LIBSVM software package for Matlab [28], [56]. MC-SVM incorporates a soft-margin approach, and uses a ‘one-against-one’ strategy [57], [58] to build a predictive model for a multi-class problem. Given a task with *m* classes (where *m*>2), the ‘one-against-one’ strategy simply constructs 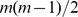 classifiers, one for each distinct pair of class labels, each learned with a soft-margin SVM. The resulting collection of 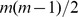 trained SVMs then operate as a single classifier via a simple voting strategy: to classify a data instance, each individual SVM is applied to the instance and the resulting class prediction counts as a ‘vote’ for that class – the predicted class is that which received the most votes, and ties are broken in favour of the class with highest frequency in the training set.

Since datasets in this field are typically unbalanced, with, for example, many more *inter-ictal* instances than *pre-ictal* instances, the ASPPR approach assigns a weight value to each class, and these weight values are exploited in the ‘one-against-one’ soft-margin SVM algorithm in the way described by Chang and Lin [28]. For a given dataset *D*, the weight values used for classes *inter-ictal*, *pre-ictal*, *ictal* and *post-itcal* were, respectively: 1, *inter_D_*/*pre_D_*, *inter_D_*/*ictal_D_*, and *inter_D_*/*post_D_*, where *inter_D_* denotes the number of *inter-itcal*, *pre_D_* denotes the number of *pre-itcal* instances, *ictal_D_* denotes the number of *ictal* instances, and *post_D_* denotes the number of *post-ictal* instances in *D.* These relative weightings effectively normalize the overall influence of each class on the predictive model.

The kernel function used in the experiments reported here was the Radial Basis Function (RBF) kernel:
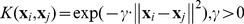
(5)where 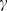 is a parameter. The RBF kernel is the most commonly employed Gaussian kernel in SVM applications; Gaussian kernels tend to be more effective than the alternative linear or polynomial kernels [59], and the RBF kernel is the default setting in the LIBSVM package [28]. Preliminary experiments were done to seek confirmation or otherwise that an RBF kernel was the appropriate choice, by building ‘*t* = 0’ predictive models for each of the 21 patients. The mean accuracies (with standard deviations in parenthesis) were 78.7% (11.7%), 96.5% (3.8%) and 97.5% (3.0%) respectively for linear, polynomial, and RBF kernels. The parameter *C* (the regularization parameter used by the SVM - equation (3)) and the parameter 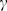 (characterizing the RBF kernel in equation (5)) are both ‘hyper-parameters’ in this context, controlling the behavior of the learning algorithm. It is customary to tune hyper-parameters in a preliminary stage. In the ASPPR method, this tuning was done separately for each individual experiment (part of the learning module in Figure 5) as described next, following the approach suggested by Chang and Lin [28]. At the start of each individual experiment (for a given patient and given time-in-advance dataset), tuning is accomplished via a grid search of combinations of (*C*, 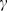) settings. Each such combination is evaluated by the Accuracy figure returned by a ten-fold cross validation run. The combination of parameters that emerge with the best performance are then used as the SVM parameters to build the predictive models. The co-ordinates of the (*C*, 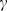) search grid were defined as follows, after preliminary testing to identify good overall regions. *C* ranged through the three values 2^8^, 2^12^, 2^16^, while 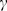 ranged through the six values, 2^0^, 2^2^, 2^4^, 2^6^, 2^8^, and 2^10^. Representative experience indicates that performance (for the RBF kernel) varies widely across this range of combinations (e.g. from ∼85% to ∼99% Accuracy), however the performance surface is smooth around the optimal region (e.g. for a typical patient, being always above 98.5% for the lower two values of 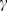, irrespective of the value of *C*). Following hyperparameter selection and construction of the Multi-class SVM, the trained predictive model is then tested on the unseen test data. The results on the test data are reported in terms of Accuracy, Sensitivity and Specificity, and a further summary *S1-*score measure, *a*s explained next.

### Evaluation Measures

ASPPR learns a predictive model that attempts to classify an unseen data instance as one of four classes: *ictal*, *pre-ictal*, *inter-ictal*, and *post-ictal*. To facilitate definition of the evaluation measures, the following notation will be useful. Referring to data instances in a test set *S* containing |*S*| instances in total, let 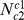 denote the number of data instances in *S* of class *c1* that were predicted to be of class *c2*, and let 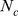
denote the total number of instances in *S* that were predicted (whether correctly or not) to be in class *c*. Hence, for example, 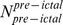 indicates the number of correctly identified *pre-ictal* instances, while 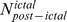 is the number of *ictal* instances that were misclassified as *post-ictal*, while 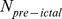 is the total number of instances from *S* predicted to be *pre-ictal.* The Accuracy figure that is reported from a single trial of ASSPR is the straightforward percentage of correct classifications on the test set. That is, Accuracy is: 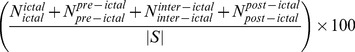
(6)


In common with the majority of prediction-oriented seizure studies, Sensitivity and Specificity are also reported, both of which are focused on the predictive model's ability to correctly classify the *pre*-*ictal* class. Sensitivity is calculated as follows, measuring the percentage of the *pre*-*ictal* class predictions that were correctly identified: 
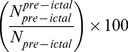
(7)


Equation (7) is equivalent to the standard definition of Sensitivity in terms of true positives (TP) and false negatives (FN), 100×TP/(TP+FN), where TP counts the number of correctly identified *pre-ictal*s and FN counts the number of instances from all other classes that were incorrectly identified as *pre-ictal*s. Meanwhile, Specificity is the percentage of the *non pre-ictal* predictions that emerged from *non pre-ictal* instances:
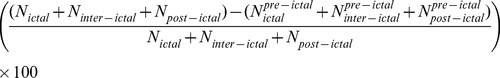
(8)


Equation (8) is equivalent to the standard definition of Specificity in terms of true positives (TN) and false positives (FP), 100×TN/(TN+FP), where TN counts the number of truly *non pre-ictal* instances that were not predicted to be *pre-ictal*, and FN counts the number of *pre*-*ictal* instances that were predicted to be from one of the *non pre-ictal* classes.

To simplify discussion of results, an *S1-score* is calculated, which is the harmonic mean of Sensitivity and Specificity, that is:
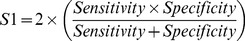
(9)where Sensitivity and Specificity are as defined above. For consistency, this measure is also reported as a percentage. This measure serves as a fair single-value summary of Sensitivity and Specificity, conservatively favouring the smaller of the two. It is similar in spirit to the *F_1_* measure that is commonly used in the field of information retrieval, which is the harmonic mean of *precision* and *recall* (where recall is equivalent to sensitivity, however precision differs from specificity).

Finally, results from a variety of baseline and ‘random predictors’ are also provided. Prediction-oriented seizure studies often omit comparison against such a baseline, despite the fact that high levels of Sensitivity and Accuracy are achievable by simple predictors under certain circumstances. For example, Winterhalder et al. [60], commenting on Chtaovalitwongse et al. [22], calculate that the majority of reported results on test patients in that study would be outperformed by a random predictor; in this case, this arises in part since the wide time horizons used by Chtaovalitwongse et al. [22] lend themselves to high Sensitivity. As a reference point for the results of ASPPR experiments in the present study, baseline predictors and random predictors are considered. The baselines are respectively predictors that always predict *pre-ictal* (indicating an advance prediction of seizure), and that always predict non *pre-ictal*. The random predictor predicts *pre-ictal* with a probability *p*, and is ‘lucky’, or unduly well-informed, in that *p* is set to the correct frequency for *pre-ictal* instances. In addition, a further ‘lucky’ random predictor, which predicts each of the four classes with the appropriate frequency, is also referred to as a baseline for Accuracy values. For these baseline and random predictors, the frequency values used for each class (in line with the frequencies in the test data) are as follows: *pre-ictal* (*p*) = 0.08333, *ictal* = 0.05, *inter-ictal* = 0.7833, and *post-ictal* = 0.08333. These values are used as the basis for comparative values of Accuracy, Sensitivity, Specificity, and S1-score.

## Results

In this section, the performance of ASPPR is characterized by reporting the results of 21 separate experiments, one for each of the 21 patients represented in the Freiburg EEG database. In addition, benchmark results were obtained by applying ASPPR to each patient, to learn the ‘*t* = 0’ predictive model, without the feature selection step, with the features instead fixed to be the 14 features used by Costa et al. [23]. Sensitivity is the primary performance measure of interest. Sensitivity values indicate precisely the ability to detect oncoming seizures – for example, Sensitivity of 90% for an individual patient reported on unseen data for that patient suggests that, in intervention, 90% of the seizures that will actually occur will be detected, and 10% of them will be missed. Deficit in Sensitivity therefore correlates with potential dangers for the patient. In contrast, deficits in Specificity mean high rates of false alarms; this is more acceptable than deficit in Sensitivity, since the intervention measure triggered by a false alarm (for example, ‘sit down for 30 minutes’) will likely be low cost and benign. Nevertheless, ideally false alarms should be minimized (hence, Specificity should be maximized) to avoid undue disruption to the patient's lifestyle. Finally, the S1-score provides a convenient summary value that simplifies comparison of results in the context of multiple patients and multiple ‘time-in-advance’ prediction regimes.

### Mean Performance Analysis

**Figure 7:**
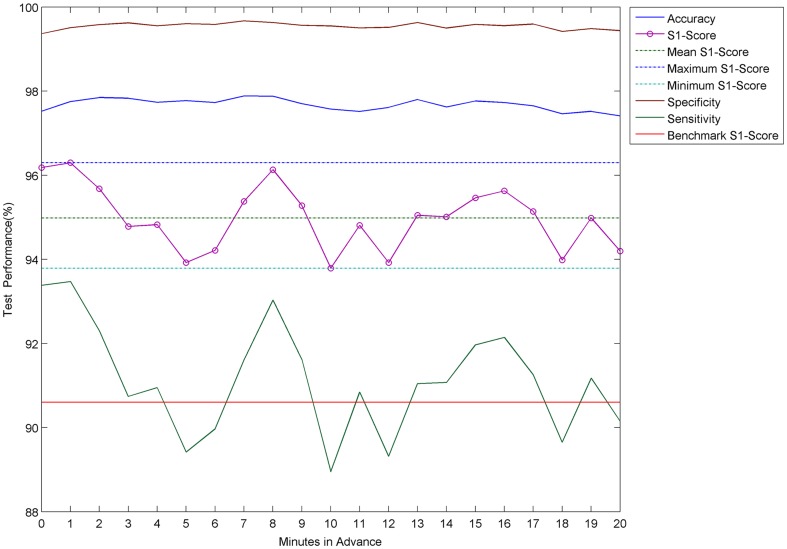
Summary results of advance prediction by ASPPR on 21 patients. The plot shows Accuracy, Sensitivity, Specificity and S1-Score averaged across all 21 patients at each prediction time-step. The plot also displays the minimum, mean, maximum and full feature-set values for the S1-Score measure as well as the benchmark S1-Score value.

Summary statistics for the performance of ASPPR are plotted in Figure 7 and also shown in Table 1. Figure 7 shows, for each of the 21 ‘time in advance’ predictive models (from ‘*t* = 0’ to ‘*t* = 20’), the mean values over the 21 patients of Accuracy, Specificity, Sensitivity and S1-score, along with the benchmark performance measure, and the overall minimum, maximum and mean of S1-score. Accuracy and Specificity lay respectively in the ranges [97.41%, 97.88%] and [99.36%, 99.67%], which are well above the benchmark. The most well-informed random predictor would achieve Accuracy of 63.0% and Specificity of 91.67%, representing the best of the baseline and random predictors (for example, the ‘always *pre-ictal*’ predictor would achieve 5% Accuracy). Sensitivity and S1-score also exhibit values that appear promising with respect to further development towards intervention treatments; however there is prominent variation between time-steps, which is discussed later. Sensitivity is within the range [88.95%, 93.47%], with a standard deviation of 1.29%. S1-Score is within the range [93.79%, 96.30%], with a lower standard deviation of 0.77%. In contrast, the Sensitivity of the well-informed random predictor would be 8.33% (with Specificity 91.67% as above, and S1-Score 15.2%); additionally, the Sensitivity of a ‘*p* = 0.5’ random predictor would be 50%, but with Specificity dropping to 50% (and S1-Score also at 50%). The S1-Score curve starts at t_0_ with 96.18% and rises a little at t = 1 to 96.30%, which is so far, the highest observed value for advance prediction, and suggests that prediction between 1 and 6 minutes in advance is more readily achievable than prediction between 0 and 5 minutes in advance. S1-score then decreases until it hits a trough at *t* = 5 with 93.92%, rises to 96.13% at t = 8, and then dips to its lowest value at *t* = 10, 93.79%. It remains at or above 95% for *t* = 13, 14, 15, and continues to stay above 94%. The red line in Figure 7 marks the benchmark S1-score of 90.6% which was produced using the default feature-set from the Costa et al. study [23]. Recall also that baseline random predictor benchmarks for S1-score are 15.2% (well-informed predictor), and 50% (‘p = 0.5’ predictor); also, an ‘always *pre*-*ictal*’ predictor would have an S1-score here of 0% (Sensitivity 100%, Specificity 0%), and a ‘never predict *pre*-*ictal*) predictor would have a similar profile, but with Sensitivity 0% and Specificity 100%. The results reveal that seizure detection and advance prediction using the ASPPR algorithm outperforms the benchmarks and baselines throughout all ‘time-in-advance’ configurations tested here.

**Table 1:** Summary of ASPPR on 21 patients.

	ACC(%)	t	SP(%)	t	SS(%)	t	S1(%)	t
**min**	97.41	20	99.36	0	88.95	10	93.79	10
**max**	97.88	7	99.67	7	93.47	1	96.30	1
**t = 0**	97.52	0	99.36	0	93.38	0	96.18	0
**t = 20**	97.41	20	99.44	20	90.15	20	94.20	20
**mean**	97.68		99.55		91.14		94.98	
**median**	97.73		99.56		91.07		95.01	
**mode**	97.41		99.36		88.95		93.79	
**std**	0.14		0.08		1.29		0.77	
**range**	0.47		0.31		4.52		2.51	

The minimum (min), maximum (max), mean, median, mode, standard deviation (std) and range of the four measures of Accuracy (ACC), Specificity (SP), Sensitivity (SS), S1-Score (S1) are listed. Additionally, the four measures at the seizure onset (*t* = 0) and at the largest prediction window (*t* = 20) have been listed. The ‘*t*’ column for each measure lists the time point of the corresponding statistical measure in minutes. E.g. the minimum value for Accuracy is at time point 20 with value 97.41%.

### Variation of Performance with Individual Patients

**Figure 8:**
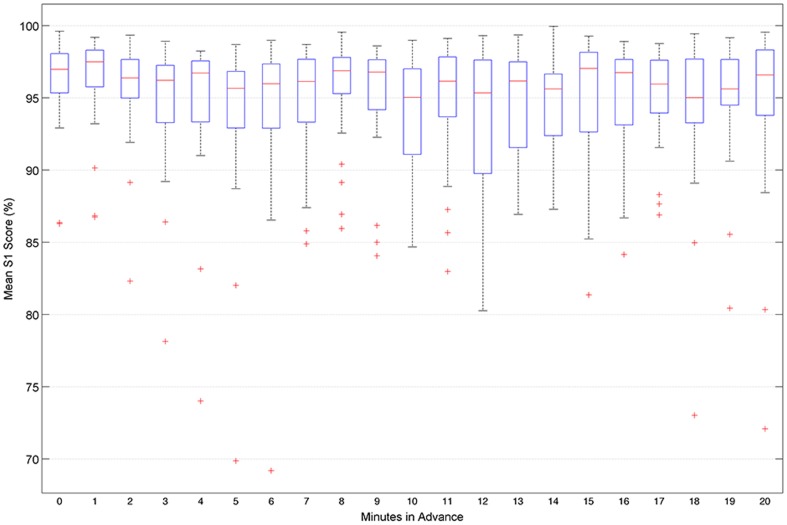
Distribution of S1-scores over individual patients for each time-in-advance prediction model. The boxes at each interval display the distribution of average S1-Score of each of the 21 patients at each advance prediction time-step.

Figure 8 shows the distribution of S1-Score for all patients across all time-in-advance prediction models. The performance measure in each box represents the distribution of *S*1-Scores over patients for the time-in-advance model indicated on the horizontal axis. The boxes indicate the higher and lower quartiles of S1-score; the length of whiskers was calculated from the inter-quartile range and the line in the middle of each box represents the median. Any points outside a box are deemed as outliers.

The most compact boxes – suggesting reliability in prediction capability - correspond to *t* = 0 (prediction of seizure between 0 and 5 minutes in advance) and *t* = 14 (prediction of seizure between 14 and 19 minutes in advance). As Figure 8 clarifies, between one and four patients, representing between approximately 5% and 20% of the cohort, tend to be outliers in one or more of the predictive models. This suggests that intervention therapy based on the approach in this paper, or similar approaches, will (like many therapies) not be suitable for everyone. It is worth noting, however, that this unsuitability would be discovered before implementation, by observing (as done here) performance of the predictive models on the patient's unseen data.

**Figure 9:**
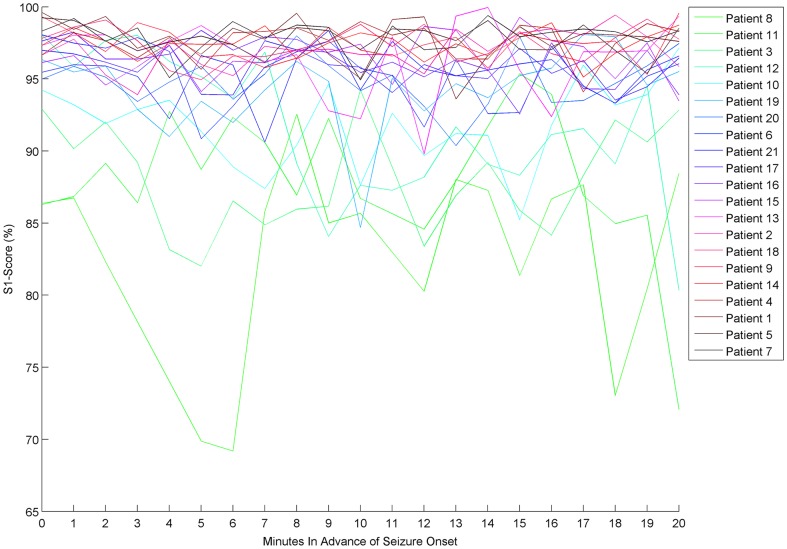
Mean S1-Score of the ASPPR algorithm for 21 patients. The legend orders the patients in ascending order of their S1-Score averaged over all time-steps.

In Figure 9 the S1-score of each patient is depicted for each time-in-advance predictive model. The S1-Score in each case is the mean of ten independent trials. The results reveal that while the majority of the patients have scores above 95% for all models, patients at the lower end of the performance spectrum generally have performance varying between 70% and 95% for all models. Moreover, the peaks and dips which were observed in the mean performance analysis (Figure 7) are not followed by all patients.

## Discussion

The body of prior work in the field of seizure prediction and detection does not seem to include studies of machine learning advance prediction that are tested and verified on a large number of patients. In earlier studies that were analysis-oriented, seizure pre-cursors were found 6 seconds prior to onset [14], [15] and 1-minute prior to onset [16], while Mormann et al. [25] evaluated 30 features in the context of various prediction windows of between 30 and 240 minutes in advance of seizure onset. These and other studies confirmed the existence of seizure markers in advance of the actual onset, but did not attempt to (or failed to) show notable predictive performance for a patient's unseen EEG data. Later prediction-oriented studies reported 68% Sensitivity and 85% specificity for predictions up to 72 minutes prior to seizure onset [22] trained and tested on 3–14 day recordings of 10 patients, and, for seizures up to 78 minutes in advance, 91% Sensitivity and 85% specificity on the continuous EEG of only two patients [21].

In this paper, ASPPR learns predictive models that are targeted directly at recognizing patterns that occur in a specific 5-minute window in advance of seizure onset. For each of 21 patients, 21 such models were learned, targeted respectively at ‘0 to 5 minutes in advance’, ‘1 to 6 minutes in advance’, and so on up to the ’20 to 25 minutes in advance’ window. Feature selection (based on the ‘0 to 5 minutes in advance’ dataset for the specific patient) was done prior to building these models, to extract the best 14 features for that patient from a total of 204 features, and these 14 were then used to build each individual predictive model via a Multi-Class Support Vector Machine, incorporating an initial hyper-parameter selection phase.

The results suggest that all of the predictive models built by ASPPR outperform both the benchmark feature set and baseline predictors. Highlights include Sensitivity of 90.15% and Specificity of 99.44% for prediction of seizure between 20 and 25 minutes in advance (means taken over unseen data for each of the 21 patients), only a small amount less accurate than the Sensitivity of 93.38% and Specificity of 99.36% for prediction of seizure onset between 0 and 5 minutes in advance.

With the summary S1-score performance measure for time-in-advance predictive models *t = *5 and *t* = 18 representing relatively low points, and time-in-advance points *t = *1 and *t* = 8 and *t = *16 exhibiting local peaks, the findings revealed an intriguing apparent periodicity with a wavelength between 7 and 8 minutes. This periodicity could be related to infraslow oscillations that are believed to be associated with sleep (which state we can tentatively assume accounts for approximately one-third of the activity recorded in the Freiburg data) and epilepsy. Vanhatalo et al. [61] demonstrated large-scale infraslow oscillations during sleep in the human cortex at wavelengths up to 50 seconds; these were observed in widespread cortical regions, and strongly synchronized with faster activities, as well as with inter-ictal epileptic events. Related recent findings include those of Ren et al. [62] who identified oscillations with wavelengths from 40 seconds to 120 seconds in three patients with refractory epilepsy occurring up to 22 minutes prior to clinical seizure onset, reflecting a dynamic process during the pre-ictal state. Ren et al termed this process ‘very low frequency oscillation’ (VLFO), and suggested it may render new insight into epileptogenesis and provide additional information concerning the seizure prediction. Meanwhile, Parri and Crunelli [63] reported calcium oscillations as slow as 0.003 Hz (corresponding to a wavelength approaching 6 minutes) generated by thalamic astrocytes. Kaiser [64] suggests that, since the astrocyte network manages energy and excitability in the thalamus, this thalamic process may influence the thalamocortical network and allow detection of corresponding infraslow frequencies in the EEG. While the oscillatory behaviour observed in Sensitivity and S1-score in Figures 7 and 8 may be partly artefact, it could arise in part from a combination of infraslow oscillatory processes (known to have large amplitude [61]), in at least some of the patients, that interact to cause complex low-frequency-dominated oscillations in the salience of signal patterns that are discriminative in the context of the predictive models.

A further issue of interest is variation in the feature-sets extracted for each patient in the first stage of building a predictive model for that patient. Variation in electrode placement, individuals, and many other conditions during the data-procurement process, make it difficult to expect high levels of overlap in the feature sets for different patients. Indeed this was seen to be the case. For example, the patient with the best mean S1-score at ‘*t* = 20’ (99.55%) was patient 9, whose feature-set comprised four instances (from different channels) of SEF measured over the LTE window, five instances of SBP features (at both STE and LTE windows), along with three Skewness (LTE) and three Mean (LTE) features. In contrast, patient 1 achieved the highest S1-Score (99.61%) for the ‘*t* = 0’ predictive model, with a feature set comprising (unlike patient 9), three of the Costa et al [23] features (two signal energy STE and one signal energy LTE) along with two instances of SEF features, six instances of SBP features, one Skewness and two instances of Kurtosis. A core of three individual features were however most common among the individual feature sets; these were SEF (LTE), Accumulated Energy, and Skewness (LTE), accounting respectively for 16.3%, 10.2% and 5.8% of the 204 ‘slots’ in the ‘top-14’ feature sets of the 21 patients. Meanwhile STE features were clearly of lower predictive value than LTE features, respectively accounting for 29.9% and 57.1% of slots, while the feature categories of SBP and SEF respectively accounted for 25.85% and 23.81% of slots. The two nonlinear dynamics features took only 1.7% of slots.

The outcome of this study contributes further evidence of advance seizure predictability. Further advances in performance may well be available in future work that explores other ways to extract discriminatory features from the EEG signals. For example, the features we use are essentially univariate and predominantly linear filters, but multivariate and other non-linear filters may have advantages in this context. It should also be noted that the feature-selection part of the comparative experiments reported here was constrained to select a feature subset of size 14, facilitating fair comparison with the benchmark. This is however unlikely to be the optimal feature-subset size, and more successful results for individual patients may well be achievable by allowing the feature-subset size to vary in an unconstrained feature selection step. This may, for example, lead to more favourable outcomes for the 20% of individuals for whom the results are currently poor. It is also worth examining the possibility of a different feature selection stage for each separate time-in-advance model (rather than, as here, one feature-set per patient). Meanwhile, we note that the promising levels of Sensitivity and Specificity in our results, combined with the fact that these results are obtained from test data in the context of relatively large numbers of both experiments and patients (in comparison with previous prediction-oriented studies), arguably bodes well for future real-world applications exploiting this technology for the benefit of epilepsy patients.
