# Correction: Predicting Epileptic Seizures in Advance

**DOI:** 10.1371/journal.pone.0119194

**Published:** 2015-03-18

**Authors:** The PLOS ONE Staff

Reference 23 is incorrect. Please refer to the correct Reference 23 here.

R. P. Costa, P. Oliveria, G. Rodrigues, B. Leitao, and D. Antonio, “Epileptic seizure classification using neural networks with 14 features,” in Proceedings of the 12th International Conference on Knowledge Based Intelligent Information and Engineering System, Part II Lecture Notes of Computer Science Series, pp. 281–288, Springer, Zagreb, Croatia, September 2008.
